# Characteristics of Soil Erodibility in the Yinna Mountainous Area, Eastern Guangdong Province, China

**DOI:** 10.3390/ijerph192315703

**Published:** 2022-11-25

**Authors:** Mingyong Zhu, Wenming He, Youcun Liu, Zhiyun Chen, Zhicheng Dong, Changbai Zhu, Yankui Chen, Yongzhu Xiong

**Affiliations:** 1 School of Geography and Tourism, Jiaying University, Meizhou 514015, China; 2 Guangdong Provincial Key Laboratory of Conservation and Precision Utilization of Characteristic Agricultural Resources in Mountainous Areas, Jiaying University, Meizhou 514015, China; 3 School of Chemistry and Environment, Jiaying University, Meizhou 514015, China

**Keywords:** soil erodibility (K), influencing factor, EPIC model, Torri model, Shirazi model, Yinna mountain

## Abstract

Soil erodibility research is of theoretical and practical significance to the prediction and prevention of regional soil erosion. At present, the study on soil erodibility in the lateritic red soil area of eastern Guangdong province is relatively lacking. Taking the forest land soil of the Yinna mountainous area as the research object, the physical and chemical properties (organic matter mass fraction, texture, moisture, bulk density, pH, aggregate content) of soil samples at different altitudes were measured with field survey sampling and indoor analysis. Soil erodibility K values were simulated with different models (the EPIC model, the Torri model, and the Shirazi model) and the regional applicability of the K simulation models was discussed. The influence of soil properties on soil erodibility was analyzed. The results showed that: (1) K values in the Yinna mountainous area are between 0.0250 and 0.0331 t·hm^2^·h/MJ·mm·hm^2^, and the K value in the subsoil layer (20–40 cm) is higher than that of the topsoil layer (0–20 cm). These values decreased significantly with the increase of altitude. The soil in the study area belongs to low–medium to medium erodible soil types. (2) The three models have certain applicability in the Yinna mountainous area, but the simulation results still lack validation. (3) Soil particle size composition is the most important factor affecting the K value in the study area. As far as the topsoil is concerned, K values increase with the increase of clay and silt content and decrease with the increase of sand content and aggregate stability. Soil erodibility has no significant correlation with pH and bulk density and has no clear relationship with the content of soil organic carbon and soil moisture. The research results can provide basic data for regional soil and water conservation and the construction of K value databases of different soil types in China.

## 1. Introduction

Soil erodibility refers to the susceptibility of soil to the detachment and transport of erosion externalities, which reflects the strength of the soil’s resistance to erosion [1], and it is commonly expressed with the soil erodibility factor, K value (the larger the K value, the more easily the soil is eroded). The study of soil erodibility is of great significance for understanding soil erosion mechanisms, quantitatively estimating soil erosion amounts, and conducting comprehensive soil erosion control measures [2]. Therefore, the research on soil erodibility has become an important topic in the soil and water conservation discipline.

Soil erodibility is an inherent attribute of soil, and its concept and evaluation are relatively complex because soil erosion susceptibility is affected by a large number of physical, chemical, biological, mineralogical, hydrological and soil profile characteristics (such as soil depth) of soil itself [3,4,5,6,7]. Soil erodibility factor (K value) is a comprehensive quantitative index, which is generally determined by field measurement, inquiring Nomograph chart and formula method. Field measurement using standard runoff plots with natural rainfall is the standard method to determine the K value, and the K value obtained with this method is often used to verify the applicability of the formula method. However, this method is time-consuming and requires a lot of manpower and material resources. The inquiring Nomograph chart needs soil structure coefficient and soil permeability grade data, which are difficult to determine, so the extension and application of this method are difficult. Studies have shown that the inquiring Nomograph chart is more suitable for temperate medium-texture soil, but not for most tropical and subtropical soils [8,9,10]. For this reason, most scholars try to solve the problem of estimating the K value of multi types of soil by establishing the empirical relationship between soil’s physical and chemical properties. Therefore, the formula method has developed rapidly in recent years. The widely used K value estimation formulas include the soil erodibility Nomograph equation, the modified Nomograph equation [11], the Shirazi model [12], the Torri model [13] and the EPIC model [14]. Some soil parameters required by the soil erodibility (modified) Nomograph equation in calculating K value are difficult to obtain, which limits their application in areas with insufficient data. The other three models only need soil particle size data and (or) soil organic carbon data to simulate the K value, thus providing a method to calculate the K value for areas lacking data.

In the study of the regional applicability of the soil erodibility model, Zhang et al. [15] and Lin et al. [16] respectively found that the uncertainty of the K value predicted with the Torri model was the least and the prediction effect was the best in the subtropical region and the Loess Plateau of China. Some studies found that the relative error between the simulation results of the EPIC model and the measured values was small, and then the EPIC model can be applied to the estimation of K values in some subtropical regions of China [17,18,19]. Wang et al. [20] and Wei et al. [21] believed that the Shirazi model was reliable and could be directly applied to the Loess Plateau of China. Zhang et al. [22] considered that the EPIC model and the Shirazi model were both suitable for the Loess Plateau of China; meanwhile, the Shirazi model had the advantage of requiring fewer parameters. The applicability of these models and their relationship in other regions (especially in areas other than the Loess Plateau) need further discussion.

Almost all soil properties can affect soil erodibility [23]. However, the key factors affecting soil erodibility are different in different regions and at different spatial scales [24], and the impact of the same factor on soil erodibility in different regions is also different. Soil aggregate stability [4,25,26,27,28,29,30], soil texture [31,32] or aggregate status [33], sand content [4], silt content [6,32] or silt content and soil structure [5], soil organic matter [32,34], each of them can become the key influencing factor of regional soil erodibility. Martinez-Murillo et al. [30] believed that soil bulk density was a good soil property for predicting the K factor. In terms of spatial scale, Wu et al. [35] believed that soil erodibility was mainly affected by clay minerals at a regional scale and soil degradation at a pedon scale, while Zhu et al. [7] believed that soil erodibility was mainly related to soil texture and organic matter content at a small scale, soil texture and pH at a medium scale, and elevation, saturated hydraulic conductivity and clay content at a large scale.

It is generally believed that soil rich in organic matter is more resistant to erosion because soil organic matter can increase the stability of soil aggregates, improve soil porosity, soil buffer capacity and anti-interference capacity, reduce soil erosion, and then reduce soil erodibility [36,37,38,39,40,41]. However, some studies have found that there were no significant correlations between soil organic matter content and soil erodibility [5,6,42,43]. Some studies reported that soil aggregate stability was an important factor affecting soil erodibility [2,25,29,44], while Bryan [45] pointed out that soil aggregation proxy cannot fully reflect soil erodibility. Some studies believed that soil clay content was an important factor affecting soil erodibility [37,46,47], while others believed that soil clay content has no effect on soil erodibility [5]. Some studies believed that soil erodibility increased with the increase in soil bulk density [23,38,48,49], while others found that there was no significant correlation between soil erodibility and soil bulk density [28]. These contradictory reports indicate that soil erodibility is relatively complex, and the impact mechanism of soil erodibility is still unclear. Currently, the research on soil erodibility in China is more on farmland than mountainous forest land. The research results cannot meet the needs of current soil erosion modeling. What kind of relationship between different soil properties and soil erodibility in the south subtropical mountainous area of China is still confused.

The objectives of this work are as follows: (1) to find out the differentiation law of soil erodibility on the altitude gradient and soil depth; (2) to reveal the applicability of mostly used soil erodibility model in the study area; (3) and to seek out the main influencing factor of soil on the soil erodibility in the study area. The research results will provide basic data for water and soil conservation and ecological construction in the study area. It has theoretical and practical significance for constructing soil erodibility databases of different soil types and predicting and preventing regional soil erosion in China.

## 2. Materials and Methods

### 2.1. Study Area

The study area (113°48′–117°11′ E, 22°30′–24°56′ N) is located in the mountainous region of eastern Guangdong province, China, which belongs to the south subtropical mountainous area of China. This region includes the whole territory of Meizhou city, some counties under the jurisdiction of Heyuan city, Shantou city and Huizhou city, with a total land area of 40 196 km^2^, of which the hilly area accounts for 81.8%. The potential risk of soil erosion in the study area is very high for its large area of steep slope land. At the same time, the climate characteristics of high temperature and rainfall, concentrated precipitation, frequent rainstorms, and hydrothermal synchronization provide a strong source of rainfall erosion. Hydrothermal synchronization causes strong weathering of rocks and minerals in this area, forming a loose and deep weathering layer and low soil corrosion resistance. Long-term and frequent human activities have caused great damage to vegetation, and large areas of slope land have been developed, which resulted in land degradation. In addition, most typhoons landing on the coast of eastern Guangdong province can produce rainstorms in the mountainous area of eastern Guangdong province, so soil erosion in this area is intense. Due to the coarse texture of erosion materials, a large amount of sediment washed away was detained nearby or along the way, resulting in serious siltation of rivers, ponds and reservoirs and deterioration of the ecological environment. Most of the studies on soil erosion in this area were qualitative descriptions and lacked research on the mechanism of soil erosion. Research on soil erodibility characteristics under the background of water and soil resources exploitation in the mountainous area of eastern Guangdong province has not been carried out. The analysis and study of soil erodibility characteristics are in line with the needs for water and soil conservation in this region.

There is a lack of long-term observation data on soil erosion in mountainous areas of eastern Guangdong province. The Yinna mountainous area is typical in the east of Guangdong province. Its soil types and soil erosion characteristics are representative in the east of Guangdong province, and so it was selected as the study area in this work. Three K estimation models (the EPIC model, the Torri model and the Shirazi model), which were widely used in soil erodibility research, were used in this study.

**Figure 1:**
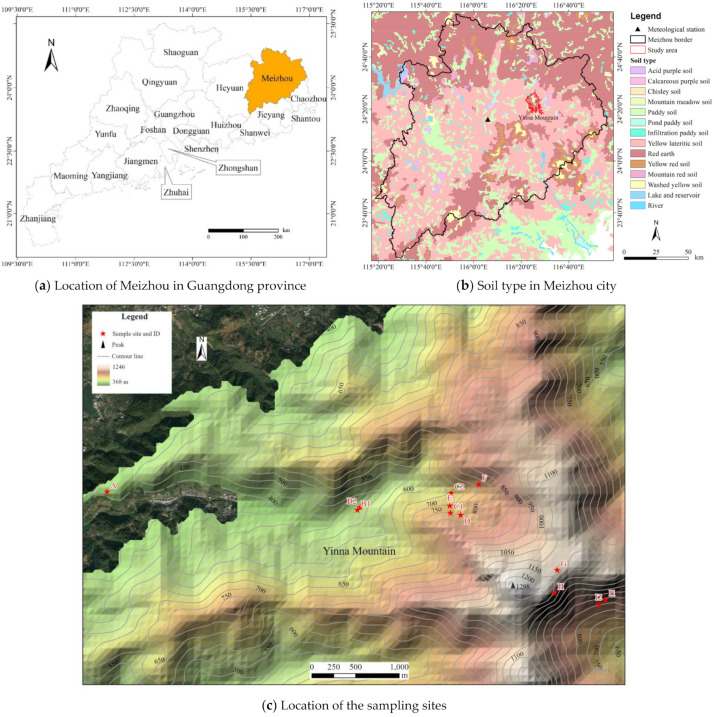
Location and soil map of the study area and sampling sites.

The area of the Yinna mountainous area (116°21′–116°25′ E, 24°21′–24°26′ N) is 6.6 km^2^ (Figure 1). The Yinna Mountain is a folded fault block mountain. The highest peak is 1297 m a.s.l. (above sea level). The mountains above 600 m a.s.l. are mostly composed of quartz sandstone, conglomerate, argillite, etc. and below 600 m a.s.l. are mainly composed of argillite, siltstone and phyllite. The region has a subtropical humid monsoon climate, with high temperature and rainy weather in summer and warm weather in winter. There is a clear distinction between the dry season and the wet season. According to the daily data of the Meijiang district meteorological observation station (Figure 1) for more than half a century, the average annual rainfall of the station is 1468 mm, mainly concentrated in the summer half year. Precipitation in the rainy season (from April to September) accounts for 74% of the annual precipitation. The average monthly rainfall is the largest in June (225 mm), and the smallest in December (32 mm). The average annual temperature is 22.2 °C. The average temperature of the coldest month in January is 12.2 °C, and the hottest month in July is 32.6 °C. The horizontal zonal vegetation of the Yinna Mountain is the subtropical monsoon evergreen broad-leaved forest, which preserves a large area of natural secondary evergreen broad-leaved forest, mainly including Fagaceae, Theaceae, and Hamamelidaceae. The zonal soil is lateritic red soil.

### 2.2. Sample Collection and Analysis

#### 2.2.1. Sample Collection

Soil samples were collected from November to December 2020, with different altitudes (accordingly, also different vegetation type) and soil layers. Starting from the base belt of the mountain, the sampling area is roughly conducted at an altitude interval of 100 m. A total of 36 sampling points are selected from 12 sampling areas (3 sampling points are arranged in each sampling area). The sampling areas should be absent any obvious human interference. The Yinna Mountain is a forest area, and the vertical differentiation of mountain vegetation is relatively obvious. From the altitude of 400 m to 1297 m a.s.l. along the elevation rise, the three-dimensional climate of Yinna Mountain will cause changes in soil, water, and heat conditions, as well as obvious vertical changes in vegetation. According to the species composition, ecological appearance, climate, and soil environment characteristics of the vegetation, it can be roughly divided into five vertical vegetation zones, and that is south subtropical evergreen broad-leaved forest, south subtropical coniferous forest, bamboo forest, evergreen broad-leaved shrub, and grass community [50]. The sampling points are roughly evenly distributed in each vegetation zone along the altitude. The sampling areas are numbered in English alphabetical order. The mountainous area is large in the foothills and near the foothills, so we increased the sampling points. The same altitude group is distinguished by subscripts. The increase of subscripts is reflected in the elevation rise within the group. The specific numbers are: A (<400 m), B_1_, B_2_ (400–500 m), C_1_, C_2_ (500–600 m), D (600–700 m), E (700–800 m), F (800–900 m), G (900–1000 m), H (1000 1100 m), I_1_, I_2_ (>1100 m). After determining the sampling area, we selected 3 vertices of a triangle with a side length of 20 m in the sampling area to sample. Samples were taken with an aluminum box and ring knife at an interval of 5 cm in the topsoil layer (0–20 cm) and subsoil layer (20–40 cm), respectively. Only 2, 7, and 4 samples can be taken from each sample point of sample areas H, I_1_, and I_2_ respectively, due to the small thickness of soil layers. A total of 255 soil samples were collected. The samples were air-dried and ground to pass through a 2-mm sieve to determine soil physical and chemical properties.

#### 2.2.2. Sample Analysis

The soil pH was determined in a 1:2.5 (weight: volume) soil to water suspension with potential method [51]. Ten grams (10 g) of pretreated soil was weighed and then placed in a 50 mL high beaker. Then fill the beaker with 25 mL pure water without CO_2_, and then seal the beaker with preservative film. Using a magnetic stirrer stir the solution vigorously for 5 min to disperse the soil particles fully, and then let it stand for 2 h. Insert the electrode (PHS-3G lightning magnetic pH meter) into the suspension of the solution and record the pH value after the reading is stable.

Bulk density was measured by ring knife method, and soil moisture was determined by drying method [51]. The sand, silt, and clay fractions in the samples were determined by the Bouyoucos hydrometer method [51].

Soil organic matter content was determined by the K_2_Cr_2_O_7_-H_2_SO_4_ digestion method [51]. Weighing 0.1 g pretreated soil which passed through 100-mesh sieve and put it into a PTFE tube and add 5 mL 0.8000 mol/L K_2_Cr_2_O_7_ standard solution in the tube, then 5 mL concentrated H_2_SO_4_, and then rotate and shake up the tube. Put the tube into a microwave digestion instrument for digestion after the solution was shaken well. After digestion, take the tube out and cool it to room temperature. Wash the mixture in the PTFE tube into a 250 mL conical flask. Then, make the volume in the flask reach 60–80 mL, add a drop of o-phenanthroline indicator, and titrate with 0.2 mol/L ferrous sulfate. The solution changes from orange yellow to blue green to brown red. Then, record the amount of FeSO_4_. Calculate the content of soil organic matter according to the amount of FeSO_4_ consumed.

Dry sieving and wet sieving methods were used to determine soil mechanical stability aggregates and water stability aggregates respectively [52]. Weigh 100 g of air dried soil passed through the 10 mm sieve and pour it into the standard soil sieve set with sieve bottom and sieve cover. A set of sieves with hole diameters of 5 mm, 2 mm, 1 mm, 0.5 mm, and 0.25 mm from top to bottom. Dry sieving the sieve set for 15 min with an 8411 electric sieve shaker. After the dry sieving was completed, the soil aggregate mass on each sieve shall be weighed and put into a self-sealing bag, and records shall be made. Finally, the mass percentage of soil aggregates of each particle size was calculated. Prepare 50 g of soil aggregates of all levels according to the mass percentage of dry sieving soil aggregate. After weighing, put it into the sieve group of the soil aggregate analyzer (TTF-100 soil aggregate analyzer). The sieve group consists of sieves with aperture of 5 mm, 2 mm, 1 mm, 0.5 mm and 0.25 mm from top to bottom. Put the sieve group of the agglomeration analyzer slowly into the iron bucket filled with water and ensure that the upper edge of the uppermost sieve will not rise to the water surface during the whole process of wet sieving. The wet sieving time is 30 min. In order to prevent the clogging of sieve openings, aggregates <0.25 mm shall not be put into the sieve group, but the content of aggregates <0.25 mm shall be included in all calculations. After wet sieving, the soil aggregates at all levels in the sieve group are washed into beakers or aluminum boxes with a washing bottle containing distilled water. Put it in an enamel tray, place it in an oven, and dry it at 105 °C for 8 h to constant weight. Cool it in the air for one day and night, weigh it and make relevant records.

Each soil sample shall be repeated three times, and the average value should be taken. The mechanical components of the soil are based on the particle composition of the American system, and the soil texture was classified. The particle size of soil aggregates was divided into >5 mm, 5–2 mm, 2–1 mm, 1–0.5 mm, 0.5–0.25 mm, and <0.25 mm. The aggregate destruction percentage (PAD) is calculated with the formula [53].

### 2.3. Calculation Method of Soil Erodibility Factor K

The widely used soil erodibility model was used to simulate the K value of the Yinna mountainous area, which includes the EPIC model, the Shirazi model, and the Torri model.

#### 2.3.1. EPIC Model

The EPIC (Erosion Productivity Impact Calculator) [14] model uses soil organic carbon and particle size composition data in the soil erosion prediction module to estimate the K value. The model structure is as follows:

(1) $$\begin{array}{l} K=\{0.2+0.3\exp[-0.0256S_{d}(1-\frac{S_{i}}{100})]\}\times (\frac{S_{i}}{Cl+S_{i}}{)}^{0.3}\times [1.0-\frac{0.25SOC}{SOC+\exp(3.72-2.95SOC)}]\\ \times [1.0-\frac{0.7SN}{SN+\exp(-5.51+22.9SN)}] \end{array}$$

where *S_d_*, *S_i_* and *Cl* (%) represent the contents of sand (0.05–2.0 mm), silt (0.02–0.05 mm), and clay (<0.002 mm), respectively. *SN*(%) = 1 − *S_d_*/100, while *SOC* (%) refers to the content of soil organic carbon derived from the measured *SOM* content (i.e., *SOC* = *SOM* × 0.58). The K value of the EPIC model calculation result is American system unit (sht·t·a·h/100 ft·sht·t·ac·int). For the benefit of using an international unit (t·hm^2^·h/MJ·mm·hm^2^), the calculated K factors are multiplied by a conversion factor (0.1317).

#### 2.3.2. Torri Model

In 1997, Torri et al. [13] proposed to establish an estimation model of erodibility using soil particle size and soil organic matter data. The Torri model is a nonlinear best-fit formula based on physical and chemical properties. Torri et al. linked the determined K values of various soils with soil properties and proposed to study the predictability and related uncertainty of K values by using soil texture parameters and organic matter content. Due to different soil texture parameter standards, unified soil texture quantitative parameters are used.

(2) $$K=0.0293(0.65-D_{g}+0.24D_{g}{}^{2})\exp[-0.0021\frac{OM}{Cl}-0.00037{\left(\frac{OM}{Cl}\right)}^{2}-4.02\mathrm{Cl}+1.72Cl^{2}]$$

(3) $$D_{g}=\sum f_{i}\mathrm{lg}\sqrt{d_{i}d_{i-1}}$$

where *D_g_* is the geometric average particle size, *OM* is the content of soil organic matter expressed as a percentage, and *Cl* is the content of clay particles (<0.002 mm) expressed by decimals. *d_i_* is the maximum value (mm) of soil particles of grade *i* in soil mechanical composition, and *d_i_*_−1_ is the minimum value (mm) of soil particles of grade *i*. When *i* = 1, then *d_0_* = 0.00005 mm. *f_i_* is the soil particle content of the corresponding particle size grade expressed by the decimal point. The geometric average particle size (*D_g_*) is calculated based on the three particle sizes of sand (0.05–2 mm), silt (0.002–0.05 mm), and clay (<0.002 mm). The Torri model can be used in the case of limited soil physical and chemical property data, and the K value in the model can be estimated based on soil particle composition. The unit of K value of this model is an international system unit.

#### 2.3.3. Shirazi Model

The formula was first proposed by Romkens et al. [54] based on 138 soil data in the United States, and then Shirazi modified the formula based on 225 soil data in the world and established the *Dg* model [12]. This model only needs to calculate the K value by geometric mean particle size. It provides a good solution to the problem of incomplete soil properties and data.

(4) $$K=0.0034+0.0405\exp[-\frac{1}{2}{\left(\frac{1gD_{g}+1.659}{0.7101}\right)}^{2}]$$

(5) $$Dg=\exp(0.01\sum f_{i}\ln m_{i})$$

where *D_g_* is the geometric average particle size (mm), *f_i_* is the percentage weight of soil particle fractions (%), and *m_i_* is the arithmetic mean value of particle size of each soil particle fraction (mm). The calculated K value is an international system unit.

## 3. Results

### 3.1. Distribution Characteristics of Soil Physical and Chemical Properties in the Yinna Mountainous Area

The physical and chemical properties of soil directly affect soil erodibility, so the analysis of soil’s physical and chemical properties is the premise and basis for evaluating the K value. Table 1 displays the soil texture and content of soil organic carbon at different altitudes in the Yinna mountainous area. The international system units were used to analyze the K value, and all the American system units were converted to international system units.

The variation ranges of pH, soil moisture, bulk density, and PAD of the topsoil layer and subsoil layer in the Yinna mountainous area and the correlation of parameters between the topsoil layer and subsoil layer are shown in Table 2.

It can be seen from Table 2 that the topsoil layer in the study area is basically neutral, while the subsoil layer is weakly alkaline, and the pH of the topsoil and subsoil layers are significantly different (*p* < 0.01). The formation of soil acidity and alkalinity is affected by natural factor and human activities. From the historical process of soil formation, the long-term spatio-temporal change of pH mainly depends on natural factors (soil material composition and geochemical properties, acid rain, etc.), but the sharp change of pH in a short time is mainly affected by human activities (such as heavy use of chemical fertilizers, urbanization, and mining development), where the diffusion of alkaline substances leads to pH increase. The Yinna Mountain is a scenic tourist area and a nature reserve with high forest coverage, so it has a strong ability to naturally adjust the soil pH value. It is far away from heavy acid rain areas (such as the Pearl River Delta region), without urbanization and mining development activities, nor does it use a large number of fertilizers, so it is less disturbed by human activities. Therefore, the neutral state of the forest soil is understandable.

According to Table 2 and the analysis, the pH and PAD of the topsoil and subsoil layers did not show significant changes with the altitude (*p* > 0.05), while soil moisture of the topsoil and subsoil layers showed a very significant increase trend with the altitude (*p* < 0.01); the topsoil layer bulk density increased significantly with the increase of altitude (*p* < 0.01), while the bulk density of the subsoil layer decreased significantly with the increase of altitude (*p* < 0.01).

#### 3.1.1. Variation of Soil Organic Carbon Content with Altitude

**Figure 2:**
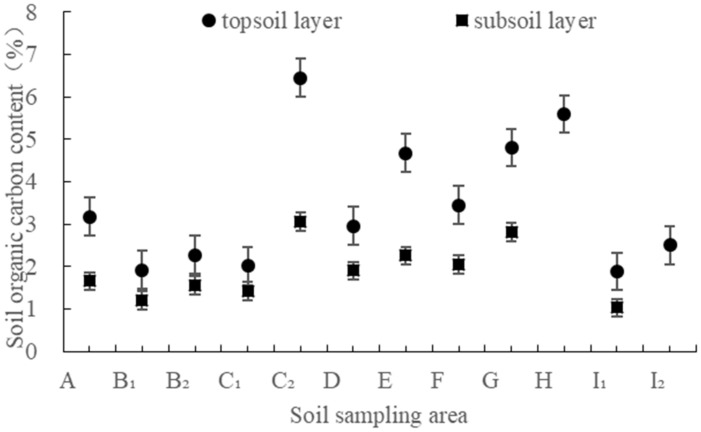
Distribution of content of soil organic carbon in the topsoil layer and subsoil layer.

The content of soil organic carbon in the study area ranges from 1.03% to 6.45%; it is 3.47% in the topsoil layer and 1.89% in the subsoil layer, respectively (Figure 2). The content of organic carbon in the topsoil layer was significantly higher than that in the subsoil layer (*p* < 0.05), and neither of them showed a significant trend of change with altitude. Nevertheless, it was evident that organic carbon content in the topsoil layer fluctuated significantly with altitude, while it fluctuated relatively little in the subsoil layer.

#### 3.1.2. Variation of Soil Particle Size in Profile with Altitude

**Figure 3:**
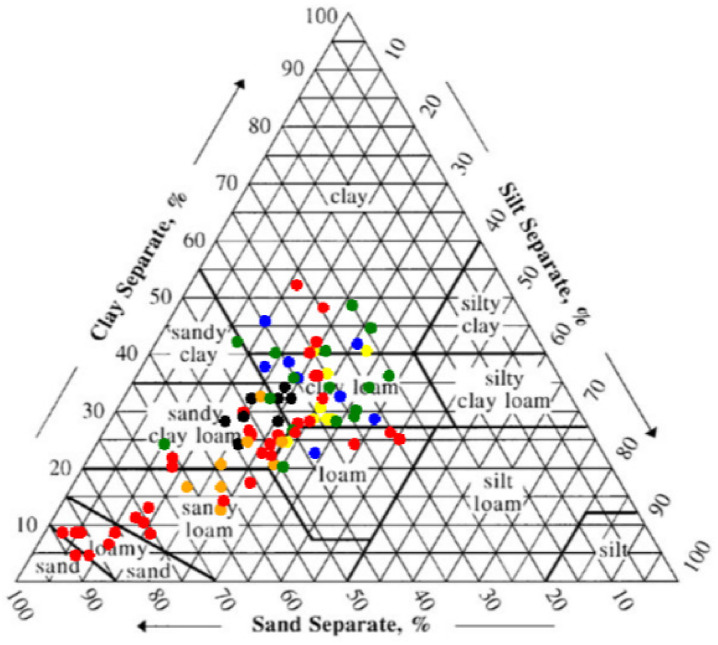
Distribution of soil texture in the Yinna mountainous area(different colors represent soil samples from different sampling areas).

Soil particle size was divided into three categories: sand (0.05~2 mm), silt (0.002~0.05 mm), and clay (<0.002 mm) according to the American system. A total of 85 soil sample values (mean value of three samples of the same depth in each sampling area is taken as a sample value) were analyzed, as shown in Figure 3 distributed in the soil texture triangle in the 12 sample areas.

**Figure 4:**
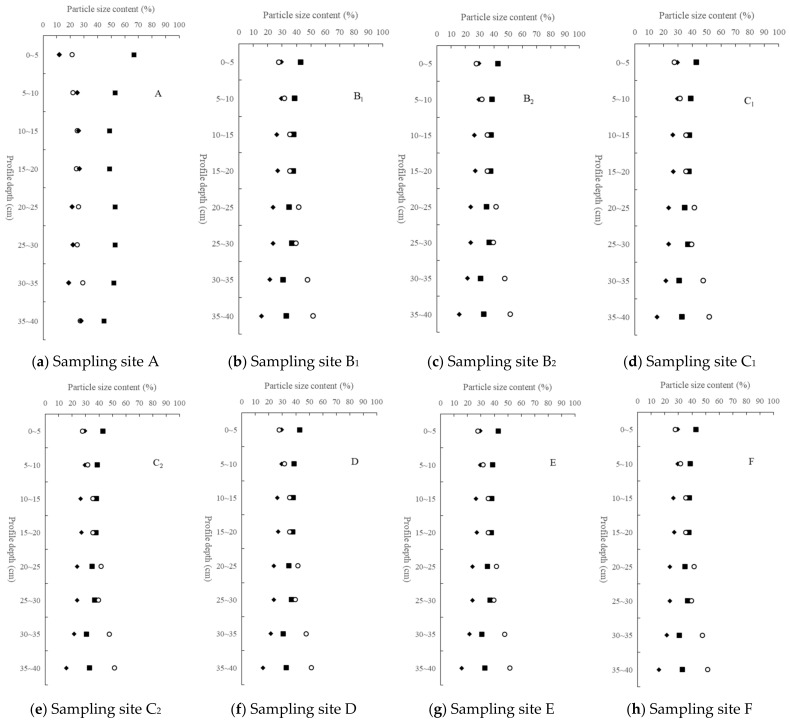

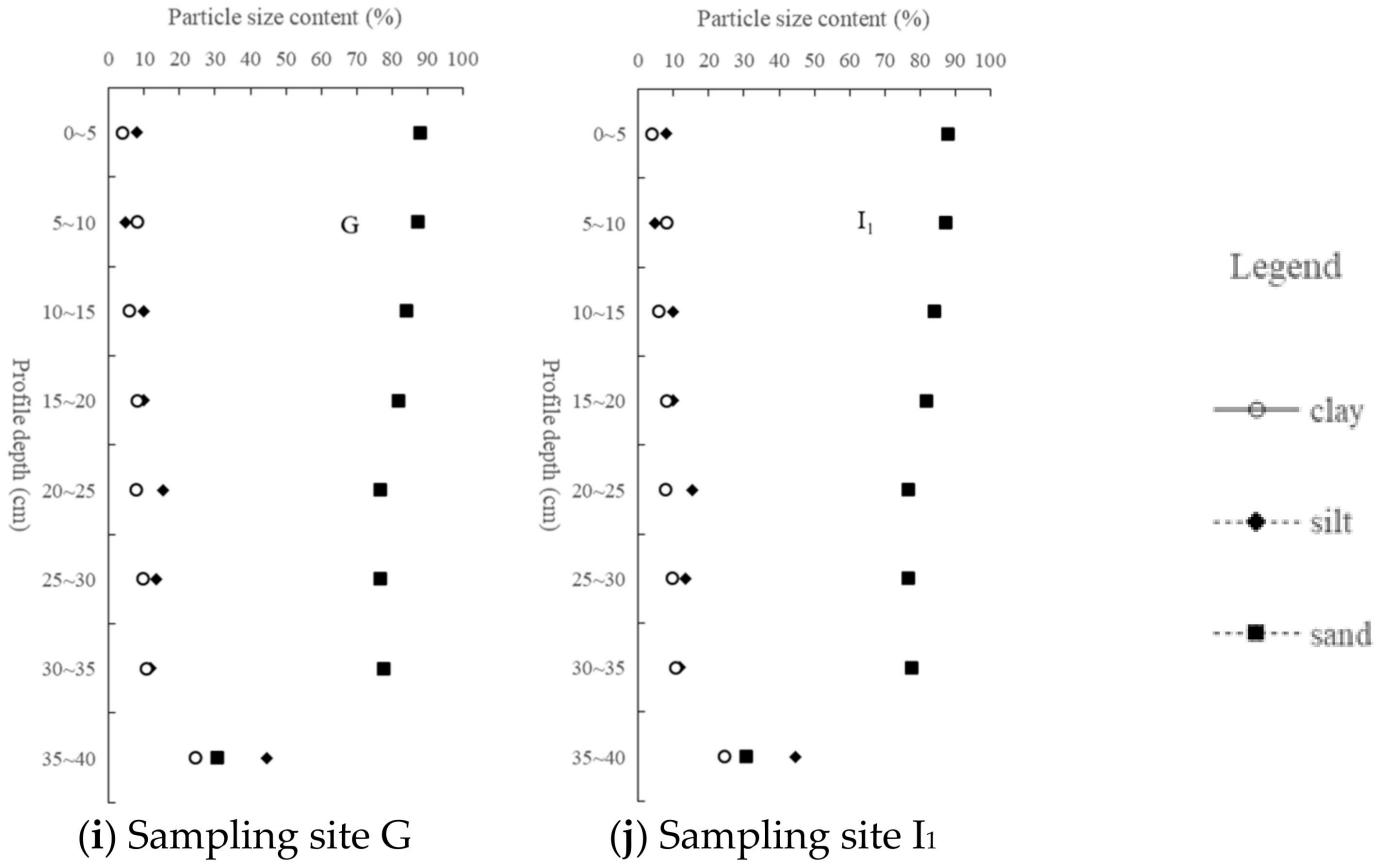
Particle size distribution of soil profiles in different soil samling areas.

It can be seen from Figure 3 that the soil texture was mainly clay loam, sandy clay loam, and sandy loam. The average content of sand (49.95%) was the highest in the study area, followed by the average content of clay (26.68%), and the average content of silt (23.36%) was the lowest, showing obvious coarse ossification characteristics. Due to the high content of sand particles, the soil particles lack cementation ability and were in a fragmented state. The ability of water and fertilizer conservation was poor, which could easily cause serious soil erosion [55]. The particle size distribution of 10 soil samples from 0~40 cm deep and a relatively complete profile are shown in Figure 4.

The soil particle size composition on the profile showed a trend of gradual increase in the content of fine particles (silt and clay) and a gradual decrease in the content of sand particles with the increase of depth (Figure 4), which was related to strong weathering and leaching in the subtropical region. The sand content accounts for the highest proportion, indicating that the soil coarseness of the Yinna mountainous area was high and the coarse sediment accounted for a large proportion.

**Figure 5:**
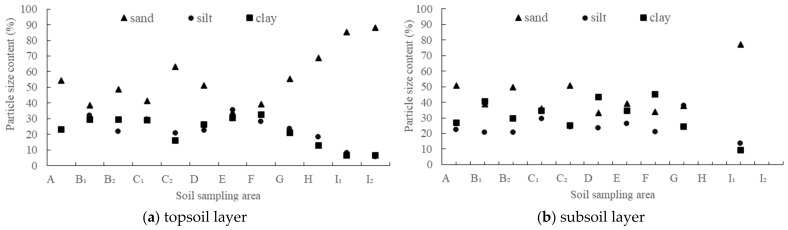
Distribution of soil particle size content at different altitudes.

As far as the depth of the soil layer was concerned, the grain size content of the topsoil layer and subsoil layer showed a very significant change trend with the increase of altitude, in which the sand content increased significantly (*p* < 0.01), and the silt and clay content decreased significantly (*p* < 0.01) (Figure 5). The soil particle size coarseness was obvious along the altitude. The slope of Yinna Mountain is relatively large, especially above 700 m a.s.l. Rainfall and runoff erosion can cause selective migration of soil particles [56]: fine particles in the topsoil layer were lost, leaving coarse particles with strong resistance to transport [57], and soil particles gradually developed towards coarsening, which is consistent with the results reported by predecessors [58,59]. Therefore, the redistribution of soil particle size has a certain indicative effect on the soil erosion process. There was no significant difference in particle size content (*p* > 0.05) between that of the topsoil layer and subsoil layer.

### 3.2. Comparative Analysis of K Value Based on Three Models

The estimated results and distribution of K mean values are shown in Table 3 according to the simulation models of Formulas (1)–(5).

**Figure 6:**
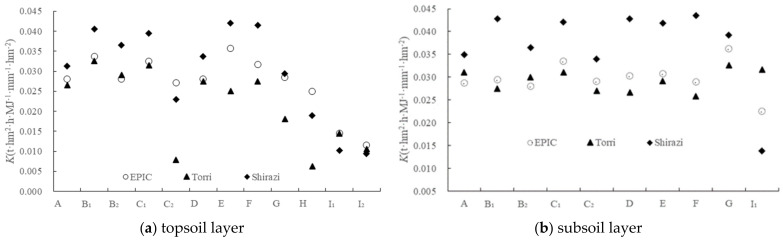
K values of different soil depths with model simulation.

The average K values of the topsoil layer and subsoil layer estimated by the EPIC model, the Torri model and the Shirazi model were 0.0116–0.0357, 0.0063–0.0325 and 0.0095–0.0420, respectively (Figure 6), and the extreme value ratios are 3.1, 5.2 and 4.4, which showed that the K values of the same profile differ greatly. The total K mean values of the topsoil layer estimated by the three models have little difference, which are 0.0270, 0.0214, and 0.0297, respectively. The K mean values of subsoil layers are 0.0298, 0.0292, and 0.0371, respectively, and the mean values of the full profile are 0.0283, 0.0250, and 0.0331, respectively. Analysis of variance showed that there was no significant difference between the K values of topsoil layers. There was no significant difference between the K_EPIC_ − K_Torri_ values of the subsoil layer, and K_Shirazi_ was significantly higher than K_EPIC_ and K_Torri_ values. Soil organic matter was involved in the K value simulation of the EPIC model and the Torri model, but not in the Shirazi model. It can be seen that under the condition of low organic matter content (it has been pointed out that the content of organic carbon in the topsoil layer was significantly higher than that in the subsoil layer), the simulation results of the models in which organic matter involved (the EPIC model and Torri model) were quite different from those in which organic matter not involved (the Shirazi model).

The K value of the subsoil layer was almost generally higher than that of the topsoil layer (Figure 2), that is to say, the K value generally increased from the topsoil layer to the subsoil layer, which was not only common in lateritic red soil areas [36,60,61,62], but also in red soil and other soil types [16,63,64,65,66,67,68,69]. With the increase in soil depth, the content of fine soil particles (silt + clay) gradually increases, and the fine particles are prone to continuous erosion by runoff, leading to an increase in soil erodibility [70]. The soil organic matter content in the topsoil layer was rich, the root density was large, and the stability of the soil structure was also a part of the reason [38,71]. This phenomenon is of great significance for water and soil conservation [26]. Once the topsoil layer with low corrosivity is damaged, it is easy to cause serious loss of the subsoil layer. Therefore, it is necessary to protect vegetation to protect the regional soil resources from loss. However, we did not find a significant difference between the K values of the topsoil layer and the subsoil layer (*p* > 0.05).

With the increase of altitude, the K value of the topsoil layer and subsoil layer showed a very significant downward trend (*p* < 0.01), which was also found in previous studies [47,72]. Altitude affects soil texture, thus affecting soil erodibility. Because there is no measured value of soil erodibility index under the guidance of a set of unified specifications in China, many studies on the estimation of soil erodibility value are still lacking in validation, especially the measured data of the K value of lateritic red soil. The K values of lateritic red soil in Fujian province, Nan’an county (in Fujian province), Guangdong province, and Sichuan province were 0.0284, 0.0286–0.0414, 0.0332, and 0.0308, respectively, based on the EPIC model simulation [62,73,74,75]. Bu et al. [76] calculated the K value of the main mountainous lateritic red soil in China as 0.0299 using the Romkens formula. Liang and Shi [77] obtained the K value of lateritic red soil in the hilly area of eastern China as 0.0282 using a nomograph chart, and Xiao et al. [78] obtained the K value of the mountainous lateritic red soil in Hainan Island as 0.0514. It can be seen that, despite the estimated K value of Xiao being obviously too large (possibly related to the fact that nomograph chart inquiry method cannot be directly applied to the tropical and subtropical regions), the K values of lateritic red soil in China estimated by different models are relatively close. According to the grading standard of soil erodibility value proposed by Liang and Shi [77], the soil in the study area belongs to medium–low to medium erodible soil.

### 3.3. Relationship between Soil Erodibility and Soil Physical and Chemical Properties

As the embodiment of soil sensitivity to erosion, soil erodibility will change with the influence of soil structure, cementation materials, etc. In order to determine the impact of influencing factors on soil erodibility, the correlation analysis of the K value and the main physical and chemical properties of soil was carried out (from the perspective of data integrity, only data of topsoil layer was picked out for correlation analysis. The study area was forest land, and soil erosion mostly occurred in topsoil layer). Pearson correlation analysis was conducted between the K value simulated by the three models and soil texture, soil organic carbon content, bulk density, pH, PAD, and soil moisture. The results (Table 4) showed that: the K value was (extremely) significantly positively correlated with soil clay and silt content and (extremely) significantly negatively correlated with sand content and PAD. The K value has no significant correlation with pH and soil bulk density. At the same time, the relationship between the K values and soil organic carbon content and the relationship between the K values and soil moisture are inconsistent. K_EPIC_ and K_Shirazi_ are more consistent, which is quite different from K_Torri_. Both K_EPIC_ and K_Shirazi_ have no significant correlation with soil organic carbon content but have a significant positive correlation with soil moisture. K_Torri_ has a significant negative correlation with soil organic carbon content but no significant correlation with soil moisture. It can be seen that soil particle size composition (including soil texture and soil aggregate composition) has become the most important influencing factor of K value in the study area.

## 4. Discussion

### 4.1. Regional Applicability of Soil Erodibility Models

The advantage of the model simulation is that the measurement of soil physical and chemical properties is mature and costs less, and the K value obtained by this method is stable and tends to the field measurement value [15], which overcomes the shortcomings of the field measurement method (it costs more) and the nomograph chart method (it requires more measurement parameters). The simulation model based on soil physical and chemical properties can more intuitively reflect the influence of these soil properties on soil erodibility. However, as for the same influencing factor, different model simulation results reflect different influences, displaying the applicability of the model.

In this work, there are no significant differences in the K values of the topsoil layer calculated by the three models, and the K values simulated by the three models exhibited (extremely) significant positive correlation. The K values simulated by the model are similar to the existing K values of lateritic red soil, which indicates that all K value models can reflect the changing law of soil erodibility to a certain extent, and it can be considered that these three models have certain applicability in the study area. However, there are differences in the accuracy of real soil erodibility information [79].

Our country lacks the measured data of the K value of lateritic red soil, so it lacks the basis to test the accuracy of simulated K value. Therefore, it is impossible to compare which K value model is more applicable in this area. Yang et al. [80] calculated the K value of lateritic red soil based on the measured data in the small area, but the result is an order of magnitude lower than the K value simulated by the formula method under normal circumstances. Because the observation period (≤5 years) of the measured data used by Yang and others is short, the K value is not only related to soil properties but also affected by rainfall and other factors. The fluctuation of rainfall during short-term observation may be large, leading to uncertainty in the calculation of the K value. Therefore, it is necessary to accumulate long-term observation data to obtain a more accurate K value as the basis for judging the regional applicability of the formula method.

Different models use different parameters, and the estimation results will also show differences. Therefore, the applicable K value estimation methods are different in different research areas [81]. The content of organic carbon (mass) was involved in the K-value calculation of the EPIC model and the Torri model, but not in the calculation of the Shirazi model. The content of organic carbon in the topsoil layer was significantly higher than that in the subsoil layer. The soil mechanical composition was involved in the simulation of the three models, and there was no significant difference between soil particle size content in the topsoil layer and the subsoil layer. There is no significant difference between the K values of the topsoil in the study area. There is no significant difference in K_EPIC_ − K_Torri_ of the sub-surface soil. K_Shirazi_ is significantly higher than K_EPIC_ and K_Torri_. Obviously, the significant difference in soil organic carbon content leads to the significant difference in simulation results of the K value of sub-surface soil. When the content of organic carbon is relatively high, the simulation results of the three models in the study area show no significant difference. When the content of organic carbon is low, the calculation results of the Shirazi model without organic carbon participation are significantly higher than those of the Torri model and Shirazi model with organic carbon participation. It can be considered that the Shirazi model has caused the loss of soil erodibility information to a certain extent, resulting in the relative increase of soil erodibility K value. In a word, it is difficult to reasonably select the estimation method of soil erodibility without the verification of measured data. If you want to obtain a more accurate K value of lateritic red soil, you need to establish runoff plots locally and further build a prediction model of the K value of lateritic red soil or optimize the existing estimation formula with the latest measured data to provide a more accurate basis for the establishment of a regional soil erosion model and water and soil loss control. The water and heat conditions in south China are good, and the difference in soil parent materials is large. Even the same soil is affected by natural conditions and soil-forming processes, and the soil properties are also different. It is necessary to accumulate longer observation data to further calibrate the K value.

### 4.2. Factors Affecting Soil Erodibility

As the object of erosion, soil mainly affects erosion by resisting the dispersion and transportation of erosion power, which determines the important role of soil physical and chemical properties (including soil texture, organic matter content, soil structure, moisture, etc.) in affecting soil erodibility [4,5,47,82,83], which is also the basis for determining soil erodibility value by the formula method [3,5]. However, the research on the impact of soil physical and chemical properties on soil erodibility is not sufficient, the indicators of soil physical and chemical properties studied are not comprehensive enough, and the research conclusions on the impact of the same physical and chemical properties on soil erodibility in different regions are inconsistent.

(1) Soil texture. Soil texture is one of the important physical characteristics of soil, which affects soil characteristics, such as water holding and aeration. The content of silt and clay in the soil is the main influencing factor of soil aeration pore [84]. With the increase of the content of clay and silt, the soil is easily transported by water, the permeability becomes poor, and the water cannot infiltrate, thus intensifying soil erosion. The sand is relatively heavy and difficult to transport. The increase of sand content in soil can improve soil structure, increase soil erosion resistance, and thus reduce soil erodibility. The stability of soil aggregates is the most important soil property affecting soil erosion [85], and its stability is an important indicator to describe the resistance of soil to external damage. Therefore, the stability of soil aggregates can be used as a stable indicator to evaluate soil erodibility [86,87].

In this work, we found that the K value of the topsoil layer simulated by the three models had a very significant negative correlation with sand content and a very significant positive correlation with silt and clay content, which was consistent with the results of most previous studies [29,62,70,88,89,90,91]. At the same time, we found a significant negative correlation between the K value and PAD, which is inconsistent with most research results [2,46,89,92,93,94]. This may be due to the strong sandy nature of the soil as a whole, which contains a lot of gravel [29].

PAD indicates the stability of soil aggregates and can directly assess soil erodibility [29,53]. K value is generally negatively correlated with the stability of soil aggregates [93]. The worse the stability of soil aggregates is, the easier the soil will be eroded. Many scholars believe that improving the stability of soil water-stable aggregates can improve soil erosion resistance and reduce soil erodibility [95,96]. Sand content can be used as an indicator of erodibility, and sand determines the crushing characteristics of aggregate water stability [4]. However, in areas with large soil sand content, whether PAD can be used as an indicator of K value needs further study.

(2) Soil organic matter. Organic matter is an important cementing material for the formation and stability of soil structure. Generally speaking, soil with high organic matter content has a strong binding force between colloids, high content of water-stable aggregates, low sensitivity to separation, high permeability [97], strong soil corrosion resistance, and low soil erodibility [2,28,39,47,69,90,93,94,98,99]. In this work, K_Torri_ has an extremely significant negative correlation with soil organic carbon (matter) content, which partially confirms the previous research conclusions. However, some studies have also found that there is no significant correlation between soil erodibility and soil organic matter [6,42,43,66], which suggests that the content of soil organic matter cannot be used as the main parameter to characterize soil erodibility [72]. Some studies even report that the K value is positively correlated with the content of soil organic matter [29,91], which is obviously inconsistent with previous understanding. Liu et al. [67] considered that the relationship between the K value and content of soil organic carbon was related to the size of soil organic carbon content and the depth of the soil layer, and a high content of soil organic carbon can maintain a constant low K value. In this work, K_EPIC_ and K_Shirazi_ showed no significant correlation with soil organic matter content. In addition to the spatial variation of soil organic matter content [5], Tejada and Gonzalez [42] considered that aspects other than content of soil organic matter, such as the chemical composition of soil organic matter and the stability of soil structure, must be considered in the calculation of K value [34].

(3) Bulk density. Soil bulk density determines the compactness, infiltration performance and water-holding capacity of the soil, which in turn affects the erosion resistance of the soil. In general, there is a negative correlation between soil erodibility and soil bulk density. Because soil with high bulk density has a solid texture, poor aeration, and poor drainage, which increase soil erosion resistance, the soil is not easily separated, leading to a decrease in soil erodibility [23,71,100,101,102,103]. In other words, the smaller the bulk density, the looser the soil, the larger the soil porosity, and the more likely it is to be lost under runoff scouring [70]. Ghebreiyessus et al. [104] found that the soil erodibility at the low unit weight (1.2 g/cm^3^) is 4.8 times that at the high unit weight (1.4 g/cm^3^). However, at the same time, the poor drainage caused by soil compaction also makes the runoff converge, and the erosion externality increase. Therefore, some studies have found the opposite model, that is, the soil erodibility increases with the increase of soil bulk density [38,39,47,48,49,83,88,98,105,106]. After the soil bulk density decreases, the soil structure is improved: the texture becomes loose, and the permeability and drainage become better, which can effectively enhance the soil permeability. The enhancement of soil infiltration capacity is beneficial to the regulation and storage of runoff, the reduction of erosion externality and the reduction of soil erodibility. Another research report says that there is no significant correlation between soil erodibility and bulk density [28,29,37,89,92,103,107], and our findings are consistent with this result. From the existing research results, the relationship between bulk density and soil erodibility is uncertain.

(4) Soil moisture. Among the soil properties that affect the soil erosion process, soil moisture is an important factor affecting not only the rate of rainfall-runoff erosion [108], but also the ability of soil to resist the separation of raindrop erosion (shear strength) and the formation of soil crust [109]. Therefore, the soil erodibility value is closely related to soil moisture. It is known that soil moisture has a complex and possibly controversial impact on soil erodibility [110]. Some studies found that K value and soil moisture were (extremely) significantly negatively correlated [47,71,100,101,105,108]: the increase of soil moisture in the early stage will enhance the degree of soil cohesion and aggregation of soil aggregates, reduce the formation of soil crusts, and soil particles will not be easily separated, and then to reduce erosion. However, the rapid wetting of dry soil will intercept air and destroy most of the binding force between soil particles, leading to the disintegration of aggregates, thus reducing the infiltration rate and significantly increasing erosion [111]. Bullock et al. [112] and Truman et al. [113] found that if soil moisture increases, soil aggregate stability will increase, and soil erodibility will decrease.

It has also been studied that the K value is (extremely) significantly positively correlated with soil moisture [88,114,115]. Kemper and Rosenau [116] and Kemper et al. [117] found that soil cohesion decreased with the increase in soil moisture. Kok and McCool [118] and Coote et al. [119] pointed out that the soil shear strength is inversely proportional to the soil moisture, which means that the soil erodibility will increase with more humid and low-strength soil conditions. Under this condition, the separation sensitivity of runoff and water drop impact increase, excessively humid conditions and low soil strength lead to reduced infiltration, and the soil is vulnerable to erosion [109,120]. When the soil moisture is low, the stable soil aggregate will increase the soil strength and reduce the separability, thus making the soil consolidated [121]. Other studies found that there was no significant correlation between soil moisture and soil erodibility [37], and our results were consistent with this report. Soil moisture can change significantly in a short time scale. In this process, the interaction between erosivity and erosion resistance of soil erosion becomes very complex. The erodibility K value of the same soil decreased, fluctuated, and increased by 1–15 times with the increase of soil moisture; the K value change of soil in a dry and wet state is also related to soil type [122]. Moragoda et al. [110] found that dry soil has the lowest resistance to erosion and thus a high erodibility, and erosion resistance increases (erodibility decreases) with increasing antecedent moisture until a certain threshold. After reaching this threshold, soil resistance decreases with a further increase in moisture, and soils become more susceptible to erosion. Therefore, soil erodibility is a complex function of soil moisture. The impact of early soil moisture on soil erodibility has not been determined, and this will become more complex due to the obvious increase of runoff caused by high early soil moisture [111].

(5) pH. The influence of pH on soil erodibility depends on the specific situation. In the soil of Ely River Valley (pH > 8.0) [47], the northern arid area of China (pH > 8.0) [107], the alkaline soil area of the Loess Plateau [99,106], and the southern acidic soil area of China (pH < 5.5) [37], researchers found that there was an extremely significant positive correlation between pH and K value. In acid soil areas, when the vegetation grows well, the decomposition of litter will produce a large number of acidic components to reduce pH, while the decomposition of a large number of litters will increase the mass fraction of soil humus, indirectly enhance the stability of soil structure, and reduce soil erodibility [37]. In alkaline soil areas, the increase in pH will lead to the imbalance of soil carbon, nitrogen, and phosphorus [123], which will inhibit the restoration of vegetation and improve soil erodibility. However, it has also been reported that pH and K values are (extremely) significantly negatively correlated [101]. Liu et al. [124] considered that the decrease in pH would destroy soil structure, reduce erosion resistance, and increase soil erodibility. Vegetation restoration can reduce soil erodibility [106]. Zhu et al. [106] and Hiradate et al. [125] believed that strong acidity (low pH) can inhibit plant growth and delay vegetation restoration, thus increasing soil erodibility. Our research found that there was no significant correlation between pH and K value, which was consistent with the research conclusion of Deng et al. [39]. Zhu et al. [106] considered that the relationship between pH and soil erodibility is related to slope: K factor in slope < 6° is positively related to pH, K factor in slope 6–25° is negatively related to pH, and pH factor is positively related to K factor in slope > 25°. It can be seen that there is an interaction between pH and environmental factors (such as slope) on soil erodibility, which makes the impact of pH on soil erodibility more complex. It is also necessary to further study the impact mechanism of pH on soil erodibility.

## 5. Conclusions

Through field sampling and indoor analysis of soil samples at different altitudes in the Yinna mountainous area, soil physical and chemical properties, such as soil mechanical composition and organic carbon content, were determined, and the soil erodibility K value was estimated using the EPIC model, the Shirazi model, and the Torri model. The conclusions are as follows:

(1) The overall mean value of K of lateritic red soil in the study area simulated by the EPIC model, the Shirazi model, and the Torri model is between 0.0250 and 0.0331 t·hm^2^·h/MJ·mm·hm^2^. The K in the subsoil layer (20–40 cm) is higher than that in the topsoil layer (0–20 cm). The K value in the topsoil layer and subsoil layer decreased significantly with the increase of altitude. The soil in the study area belongs to medium–low to medium erodible soil.

(2) The EPIC model, the Shirazi model, and the Torri model have certain applicability in the Yinna mountainous area, but the model that only uses soil particle size to simulate K value (the Shirazi model) may lead to a loss of soil erodibility information. The simulation results of the three models still need to be validated.

(3) Soil particle size composition is the most important factor affecting soil erodibility in the study area. As for the topsoil layer, the K value increases with the increase of soil clay and silt content and decreases with the increase of soil sand content and soil aggregate stability. There is no remarkable correlation between soil erodibility and pH, and there is also no significant correlation between soil erodibility and soil bulk density. However, the relationship between soil erodibility, soil organic carbon, and soil moisture is unclear and needs further research.

## Figures and Tables

**Table 1:** Soil physical and chemical properties of different altitude zones in the Yinna mountainous area (%).

	Topsoil Layer (0–20 cm)				Subsoil Layer (20–40 cm)			
Soil Sampling Area	Sand (0.05–2 mm)	Silt (0.002–0.05 mm)	Clay (<0.002 mm)	Organic Carbon	Sand (0.05–2 mm)	Silt (0.002–0.05 mm)	Clay (<0.002 mm)	Organic Carbon
A	54.3	22.5	23.2	3.17	50.6	22.5	26.9	1.65
B_1_	38.6	32.1	29.3	1.92	38.8	20.8	40.4	1.20
B_2_	48.8	21.9	29.3	2.27	49.6	20.8	29.6	1.56
C_1_	41.3	29.7	29	2.01	36	29.5	34.5	1.42
C_2_	63.3	20.7	16	6.45	50.7	24.3	25	3.06
D	51.3	22.6	26.1	2.96	33.15	23.45	43.4	1.91
E	34	35.7	30.3	4.68	39.1	26.3	34.6	2.26
F	39.3	28.1	32.6	3.45	33.8	21.1	45.1	2.05
G	55.6	23.5	20.9	4.80	37.8	37.9	24.3	2.81
H	68.8	18.2	13	5.59				
I_1_	85.3	8.2	6.5	1.88	77.1	13.5	9.4	1.03
I_2_	88.0	5.5	6.5	2.51				

**Table 2:** Statistical characteristics of some soil parameters in the Yinna mountainous area.

	pH	Soil Moisture	Bulk Density (g·cm^−3^)	PAD
Topsoil layer	6.86–7.35	4.93–78.96%	0.90–1.36	2.33–9.44%
Subsoil layer	7.13–7.56	9.42–75.30%	0.92–1.56	6.04–10.39%
Correlation of topsoil and subsoil parameters	Very significant difference (*p* < 0.01)	No significant difference (*p* > 0.05)	No significant difference (*p* > 0.05)	No significant difference (*p* > 0.05)

**Table 3:** K Values simulated by the EPIC model, the Torri model and the Shirazi model.

Soil Sampling Area	K_EPIC_		K_Torri_		K_Shirazi_	
	0–20 cm	20–40 cm	0–20 cm	20–40 cm	0–20 cm	20–40 cm
A	0.0280	0.0287	0.0265	0.0310	0.0312	0.0349
B_1_	0.0337	0.0295	0.0325	0.0274	0.0406	0.0428
B_2_	0.0281	0.0280	0.0290	0.0300	0.0365	0.0365
C_1_	0.0325	0.0335	0.0315	0.0311	0.0395	0.0421
C_2_	0.0271	0.0291	0.0079	0.0270	0.0230	0.0340
D	0.0281	0.0302	0.0274	0.0267	0.0337	0.0428
E	0.0357	0.0308	0.0250	0.0291	0.0420	0.0418
F	0.0316	0.0289	0.0274	0.0258	0.0415	0.0435
G	0.0285	0.0363	0.0181	0.0325	0.0294	0.0392
H	0.0250	-	0.0063	-	0.0189	-
I1	0.0145	0.0225	0.0145	0.0317	0.0102	0.0138
I2	0.0116	-	0.0106	-	0.0095	-

**Table 4:** Correlations between K value and soil physical and chemical properties.

	K_EPIC_	K_Torri_	K_Shirazi_	Organic Carbon	Sand	Silt	Clay	pH	Soil Moisture	Bulk Density
K_Torri_	0.647 *									
	0.023									
K_Shirazi_	0.938 **	0.841 **								
	0.000	0.001								
Organic carbon	0.183	−0.602 *	−0.100							
	0.569	0.038	0.757							
Sand	−0.968 **	−0.788 **	−0.993 **	0.015						
	0.000	0.002	0.000	0.967						
Silt	0.984 **	0.678 *	0.942 **	0.115	−0.962 **					
	0.000	0.015	0.000	0.751	0.000					
Clay	0.905 **	0.855 **	0.994 **	−0.138	−0.966 **	0.859 **				
	0.001	0.005	0.000	0.704	0.000	0.001				
pH	0.360	0.489	0.460	−0.302	−0.446	0.404	0.456			
	0.306	0.152	0.181	0.396	0.196	0.247	0.186			
Soil moisture	0.703 *	0.370	0.641 *	0.074	−0.691 *	0.776 **	0.562	0.360		
	0.023	0.292	0.046	0.839	0.027	0.008	0.091	0.307		
Bulk density	−0.340	0.422	−0.050	−0.860 **	0.134	−0.283	0.017	0.133	−0.117	
	0.336	0.225	0.891	0.001	0.713	0.429	0.963	0.714	0.748	
PAD	−0.753 *	−0.711 *	−0.854 **	0.191	0.847 **	−0.785 **	-0.846 **	−0.505	−0.669 *	−0.213
	0.012	0.021	0.002	0.598	0.002	0.007	0.002	0.137	0.034	0.555

Note: * Significant correlation (*p* < 0.05), ** Extremely significant correlation (*p* < 0.01).

## Data Availability

Not applicable.
